# Extensional Rheology of Poly(vinylidene fluoride)/N,N-dimethylformamide Solutions

**DOI:** 10.3390/polym15051119

**Published:** 2023-02-23

**Authors:** Lei Xu, Mingxiang Ju, Wentai Guo, Shengrui Yu

**Affiliations:** School of Mechanical and Electronic Engineering, Jingdezhen Ceramic University, Jingdezhen 333403, China

**Keywords:** Trouton ratio, extensional viscosity, shear viscosity, uniaxial extension

## Abstract

Typical extension flow occurs in electrospinning process of Poly(vinylidene fluoride) (PVDF) solutions such that researchers focus on extensional rheological behaviors of PVDF solutions. The extensional viscosity of PVDF solutions is measured to know the fluidic deformation in extension flows. The solutions are prepared by dissolving PVDF powder into N,N-dimethylformamide (DMF) solvent. A homemade extensional viscometric device is used to produce uniaxial extension flows and the feasibility of the viscometric device is verified by applying the glycerol as a test fluid. Experimental results show that PVDF/DMF solutions are extension shinning as well as shear shinning. The Trouton ratio of thinning PVDF/DMF solution is close to three at very low strain rate and then reaches a peak value until it drops to a small value at high strain rate. Furthermore, an exponential model may be used to fit the measured values of uniaxial extensional viscosity at various extension rates, while traditional power-law model is applicable to steady shear viscosity. For 10~14% PVDF/DMF solution, the zero-extension viscosity by fitting reaches 31.88~157.53 Pa·s and the peak Trouton ratio is 4.17~5.16 at applied extension rate of less than 34 s^−1^. Characteristic relaxation time is *λ*~100 ms and corresponding critical extension rate is ${\dot{\varepsilon }}_{c}$~5 s^−1^. The extensional viscosity of very dilute PVDF/DMF solution at very high extension rate is beyond the limit of our homemade extensional viscometric device. This case needs a higher sensitive tensile gauge and a higher-accelerated motion mechanism for test.

## 1. Introduction

Poly(vinylidene fluoride) (PVDF) products have been widely applied in petrochemical field as they posses great chemical resistance to prevent themselves from chemical attack [1,2]. PVDF material may also prevent prolonged corrosion under harsh environmental conditions such that it is frequently used to fabricate outdoor products [3,4]. More applications are made to electrical equipment and electronic devices by means of good electrical insulation of PVDF [5,6]. 

In order to manufacture high-quality PVDF products, many studies focused on the production processes, which include spinning, injection molding, blow molding, and foaming, and so on [7,8]. In these processes, PVDF melts or PVDF solutions undergo an obvious extensional phenomenon in which fluid elements are stretched without being sheared along the flow direction. For example, uniaxial extension flows occur in spinning processes, where axisymmetric fluid is extended in the axial direction and contracted in the radial direction. Biaxial extension flows arise in injection molding, blow molding, and foam molding processes [9], where polymer melts are extended in two directions and compressed in one direction. These extension flows commonly goes through an entrance, an orifice, a stagnation point, and so on. In many cases, polymer fluids behave as a mixed flow that combines the characteristics of extension and shear flows. 

Some researchers take an interest in the extensional behavior and stretching effect of PVDF melts. Sadeghi et al. [10] observed that PVDF resin of high molecular weight is strongly strain-hardening, which indicates that extensional stress increases with increasing strain above the yield point, after an initial elastic region during a cold stretching stage. Hedhli et al. [11] showed that PVDF melts at 180 °C are strain-hardening as the measured values of extensional viscosity at extension rates of 0.5, 5, and 20 s^−1^ are higher than the calculated values of steady-state viscosity in the Newtonian region. Guo et al. [12] prepared PVDF fibers by melt spinning, in which a cold drawing process may convert 80% *α* phase crystal structure into *β* phase and even the incorporation of a trace of amino-modified double wall carbon nanotube could further enhance the *β* phase content. High *β* phase content results in high piezoelectricity of PVDF melt. Bairagi and Ali [13] fabricated a piezoelectric nanogenerator to supply wearable electronic goods with the energy by melt-spinning and the nanogenerator was made of melt-spun PVDF/KNN nanorods containing 26% *β* phase content. 

Other researchers pay attention to the extensional effect of PVDF solutions in recent years. It is found that electrospun PVDF nanofibers also improve the piezoelectricity by enhancing the polar *β* phase content of polymorph polymer [14,15,16]. Although the enhancement mechanism is not very clear, polymeric molecules must be highly extended at very high draw ratio during the stretching process and polarized in the presence of extremely non-uniform electrostatic field, which is a key characteristic of the electrospinning. Electrospun PVDF nanofibers may be applied to piezoelectric films [17,18], lithium-ion battery diaphragms [19,20], and waterproof breathable films [21,22]. Many methods are developed to make composite PVDF fibers by electrospinning [23,24]. For example, Du et al. [25] electrospun highly oriented PZT/PVDF porous fibers and constructed a multi-layered piezoelectric nanogenerators via a laminating method. Tian [26] produced PDMS/PVDF fibrous membranes for oil-water separation, whose efficiency reaches 98.7% when the ratio of PDMS to PVDF is 1:2, by electrospinning. Lyu et al. [27] fabricated grapheme oxide doped PVDF/CuO/Al nanocomposites via electrospinning process, which improved the heat of reaction and anti-oxidation capability. 

These growing applications cause a concern over the extensional rheological behavior of PVDF solutions during an electrospinning process. Furthermore, the extensional rheological behavior of solutions may be characterized by an extensional viscosity and the Trouton ratio defined as the ratio of extensional viscosity to shear viscosity. Typically, the extensional viscosity and Trouton ratio are constants for Newtonian fluids, but they are variable and complex for non-Newtonian fluids. Ng et al. [28] demonstrated that aqueous grapheme oxide solutions have a Nowtonian-like fluid behavior in uniaxial extension at a volume concentration of less than 0.075 vol. % and an extension-thinning behavior with the Trouton ratios nearly independent of deformation rates at a concentration of larger than 0.1 vol. %. Yuan et al. [29] illustrated that okra mucilage is extension-thinning as well as shear-thinning, but the Trouton ratio increases monotonically with increasing strain rate. Ochowiak et al. [30] showed that polyacrylamide solutions with a mixed solvent of glycerol and water are extension-thickening and shear-thinning. The Trouton ratios also increase monotonically. 

In this work, we investigate the extensional rheology of PVDF solutions to know well the deformation and flow of matter in a stretching process. Commercially, the viscometric technology for the extensional viscosity of dilute solutions is not mature by comparison with traditional shear rheometry, though some laboratory apparatuses are developed for polymer melts to produce extension flows [31,32,33]. Thus, a homemade extensional viscometric device is applied to produce a uniaxial extension flow and test the extensional viscosity. A known Newtonian fluid, i.e., glycerol, is used as a standard test solution to evaluate the feasibility of the extensional viscometric device. The Trouton ratio of PVDF solutions is further discussed in conjunction with steady shear viscosity. 

## 2. Theoretical Background

### 2.1. Extensional Viscosity

**Figure 1:**
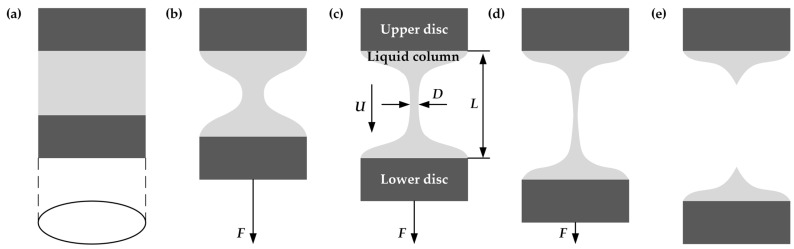
A typical stretching process of PVDF/DMF solution under applied pull force *F*: (**a**) the cylindrical shape at rest, (**b**) the waist shape at an early stage of stretching, (**c**) typical liquid column during stretching, (**d**) slender filament before breaking off, and (**e**) pinch-off.

Figure 1 illustrates a schematically stretching process of PVDF/DMF solution. Initially, the solution is placed between a stationary upper disc and a moveable lower disc (Figure 1a) to form a liquid column during stretching process. With the downward movement of lower disc, the minimum diameter *D* of liquid column at medium height decreases while the length *L* of liquid column increases. The pull force *F* increases rapidly to a peak value under applied large cross-section liquid column. Liquid column necks down after tensile deformation (Figure 1b). Subsequently, liquid column becomes finer and finer such that pull force decreases gradually (Figure 1c). Liquid column then becomes a long filament until it breaks off (Figure 1d). Pull force vanishes once the filament is broken (Figure 1e). 

In such stretching process, polymer solution forms a uniaxial extension flow and liquid column is subject to the pull force, surface tension force, and gravity. The uniaxial extensional viscosity *μ_E_* of polymer solution is defined by

(1) $$\mu_{E}=\frac{N_{1}}{\dot{\varepsilon }},$$

where *N*_1_ is the first normal stress difference and $\dot{\varepsilon }$ is the extension rate. The extension rate is naturally a velocity gradient along flow direction and expressed as

(2) $$\dot{\varepsilon }=\frac{du}{dz},$$

where *u* is the transient flow velocity. The first normal stress difference is the difference between axial stress and radial stress on a fluidic element in an axisymmetric flow. Assuming that inertial effect is ignored owing to small acceleration, we obtain the first normal stress difference by [34]

(3) $$N_{1}=\frac{4F}{\pi D^{2}}-\frac{2\sigma }{D}-\frac{\rho gL}{2},$$

where, *σ* is the surface tension coefficient, *ρ* is the density, and *g* is the gravitational acceleration.

### 2.2. Shear Viscosity

Generally, the shear viscosity *η* of polymer solutions is in line with the power-law model

(4) $$\eta =K{\dot{\gamma }}^{n-1},$$

where *K* is the consistency coefficient,$\;\dot{\gamma }$ is the shear rate, and *n* is the power-law index. The power-law index characterizes the degree of non-Newtonian behavior. The value of index is *n* = 1 for Newtonian fluids, *n* > 1 for dilatant or shear-thickening fluids, and *n* < 1 for pseudo-plastic or shear-thinning fluids. Most polymer melts possess the pseudo-plasticity with the index between 0.2 and 0.7. 

### 2.3. Trouton Ratio

The Trouton ratio *T_r_* is expressed as [35]

(5) $$T_{r}=\frac{\mu_{E}\left(\dot{\varepsilon }\right)}{\eta \left(\dot{\gamma }\right)}.$$

The formula indicates that the Trouton ratio has a dependency on extension rate or shear rate, but the extension rate is independent of the shear rate in fact. To remove the ambiguity, Jones et al. [36] calculated the Trouton ratio for uniaxial extension flow at an effective shear rate of

(6) $$\dot{\gamma }=\sqrt{3}\dot{\varepsilon }.$$

Thus, Equation (5) is transformed into

(7) $$T_{r}=\frac{\mu_{E}\left(\dot{\varepsilon }\right)}{\eta \left(\sqrt{3}\dot{\varepsilon }\right)}.$$

For uniaxial extension flows, the Trouton ratio is *T_r_* = 3 for Newtonian fluids. Specially, it is close to three at very low strain rates for polymer fluids [37]. For planar extension flows, on the other hand, the effective shear rate is displaced by $\dot{\gamma }=2\dot{\varepsilon }$ [38] and the Trouton ratio is *T_r_* = 4 for Newtonian fluids [39]. The Trouton ratio for Newtonian fluids is often used as a reference value to evaluate the feasibility of a new device designed for the measurement of extensional viscosity. 

## 3. Solution Preparation and Experiments

### 3.1. Solution Preparation 

PVDF is non-polar polymer and may be dissolved in the solvent such as N,N-dimethylformamide (DMF), N,N-dimethylacetamide, and dimethylsulfoxide [40]. Applied PVDF solutions were prepared by adding PVDF powder into DMF little by little for slow dissolution. Table 1 lists the commercial information of PVDF and DMF. PVDF/DMF solutions were sealed with tin foil in a beaker and then stirred by a magnetic stirrer (Joanlab HS-12) at 60 °C for 2 h until the solution became transparent. The concentration of PVDF/DMF solutions was 10%, 12%, and 14% by weight as they were frequently used in electrospinning process. 

### 3.2. Experimental Methods

**Figure 2:**
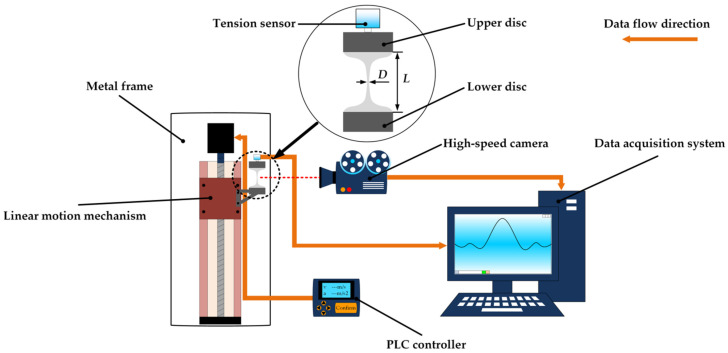
A homemade extensional viscometric device.

The extensional viscosity of PVDF/DMF solutions was measured by a homemade extensional viscometric device using a stretching method of constant extension rate as shown in Figure 2. Two discs with the diameter of 50 mm were used to hold PVDF/DMF solution. The upper disc was fixed on a metal frame and the moveable lower disc was attached to a linear motion mechanism, which was driven by a servo motor (Shanghai Yibiao DKG-Y110) via a PLC controller. A tension sensor (FUTEK LSB200) was fastened on the upper disc to test the pull force. The stretching process of PVDF/DMF solution was recorded at 211 frames per second using a high-speed camera (Hengxin AZ9501) with an image processing software (AMCap). The homemade extensional viscometric device may produce a uniaxial extension flow for test at a constant extension rate when the lower disc moved at a constant acceleration. One stretching experiment got one measured value of extensional viscosity. 

The shear viscosity of PVDF/DMF solution was measured by a commercial rheometer (Guangzhou Biuged in China, BGD 157) using a concentric cylinder or circular Couette geometry. It produced a steady shearing flow in a material cup when a rotor rotates at a constant angular speed. The flow relation between shear viscosity and shear rate was tested by adjusting the rotor speed of shear rheometer to change the shear rate. 

**Figure 3:**
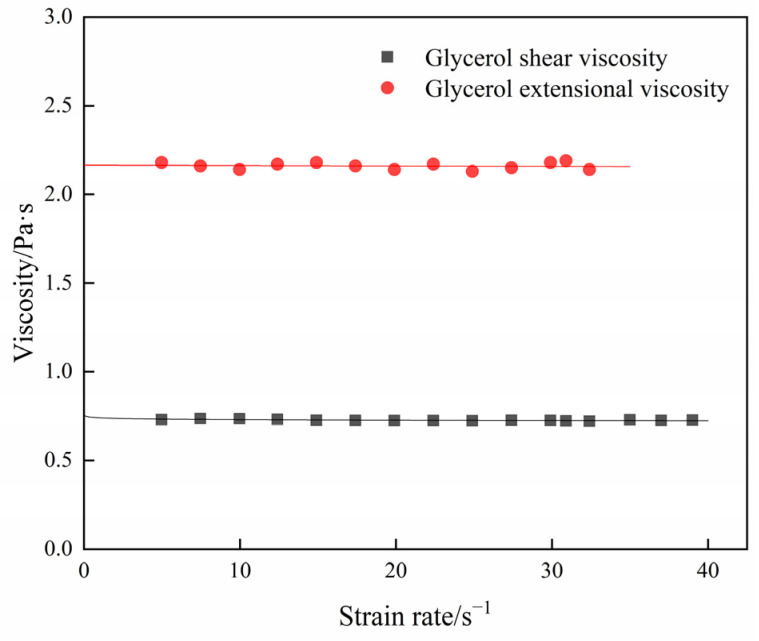
Rheological properties of glycerol.

A trial was then carried out to verify the feasibility of homemade extensional viscometric device by using the glycerol as a standard solution. Approximately 1 mL of glycerol was loaded between two discs and the extensional viscosity was measured by the homemade device. The shear viscosity of glycerol was measured by shear rheometer and the Trouton ratio was calculated by Equation (7). Figure 3 shows the steady rheological properties of glycerol. The extensional viscosity and shear viscosity of glycerol are about 2.15 Pa·s and 0.72 Pa·s, respectively, and the Trouton ratio is 3 by Equation (7). The viscosities and Trouton ratio are independent of strain rate. The result is in accord with theoretical analysis for a Newtonian fluid. It indicates that the homemade extensional viscometric device is practical and accurate. 

## 4. Results and Discussion

### 4.1. Tensile Evolution of PVDF/DMF Solutions 

Applied PVDF/DMF solution is held between discs and becomes a liquid column with an initial diameter of 18 mm and an initial length of 2 mm. During the stretching process, PVDF/DMF solution flows along the extensional direction. Liquid column grows longer and thinner under applied pull force while long molecular chains of polymer are stretched in the solution. Viscous resistance to extension flow results from the entanglement of macromolecular chains [14] and increases internal friction of the solution. Usually, viscous resistance to extension flow is higher than one to shear flow for polymer solution. Liquid column pinches off as tensile stress exceeds viscous stress and tensile strength of PVDF/DMF solution. 

**Figure 4:**
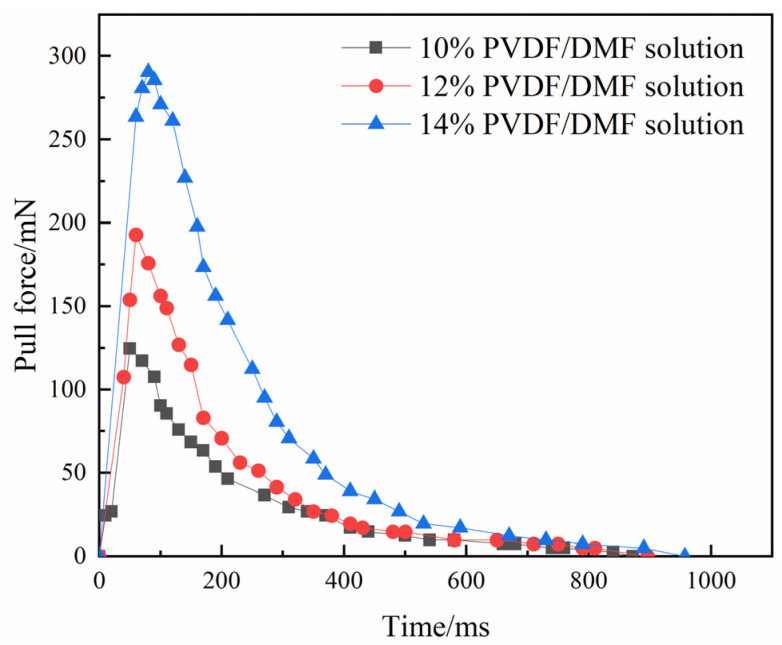
Pull force versus stretching time.

Figure 4 demonstrates pull forces exerted on PVDF/DMF solutions when lower disc moves at an acceleration of 200 mm/s^2^. Pull force changes with stretching time. Initially, pull force rises steeply to a peak at a stretch. The peak value is 124.51 mN at 50 ms for 10% PVDF/DMF solution, 192.87 mN at 60 ms for 12% PVDF/DMF solution, and 290.53 mN at 80 ms for 14% PVDF/DMF solution. Subsequently, pull force decays exponentially with time. 

It indicates that the distribution of pull force in time may be divided into a short-lived linear stage and a following nonlinear stage. Naturally, the initial linear stage results from a response lag of pull force or the time lag between input and output signals. The viscous effect of PVDF/DMF solution dominates the non-linear stage in which the pull force may be fitted with an exponential function. On the other hand, the peak pull force depends strongly on polymer concentration. High concentration of polymer solution needs high pull force to overcome flow resistance.

**Figure 5:**
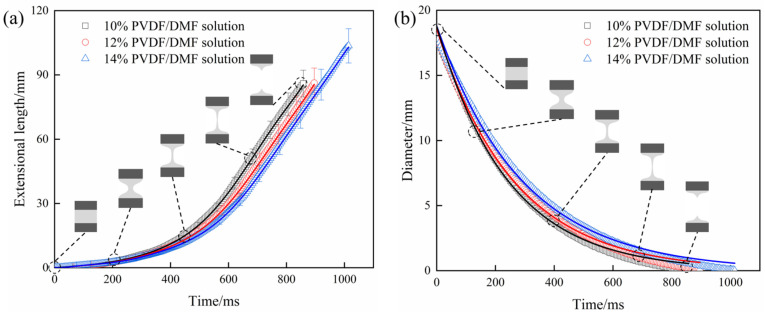
The size of PVDF/DMF liquid column: (**a**) extensional length and (**b**) minimum diameter at medium height.

As the pull force is applied, liquid column also varies during stretching process. Figure 5 depicts the size of liquid column when lower disc moves at an acceleration of 200 mm/s^2^. With stretching time, the length of liquid column increases (Figure 5a) while the minimum diameter of liquid column at medium height decreases (Figure 5b). Liquid column breaks off as the length reaches 86 mm at 856 ms for 10% PVDF/DMF solution, 86.24 mm at 896 ms for 12% PVDF/DMF solution, and 103.59 mm at 1014 ms for 14% PVDF/DMF solution. 

**Figure 6:**
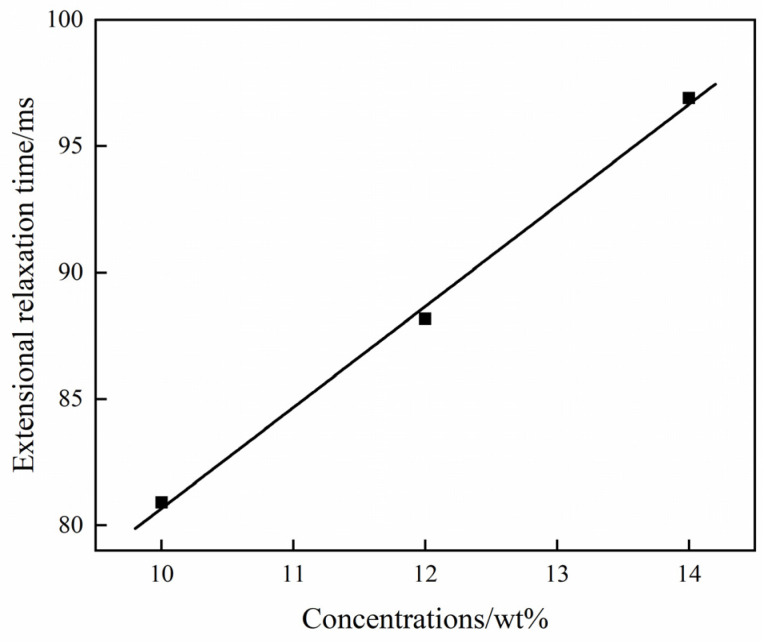
Extensional relaxation time versus polymer concentration.

Unlike discontinuous force-time curves, the length-time and diameter-time curves of liquid column are smooth since uniaxial extension flow with radial contraction is continuous. Furthermore, the thinning of liquid column may characterize an extensional relaxation process of PVDF/DMF solution. Usually, relaxation means a recovery from a perturbed system, e.g., a heat system [41], into equilibrium. The extensional relaxation is naturally a stress relaxation in company with the return of polymer chains from an extended state to a coiled state. The time evolution of the minimum diameter *D* of liquid column is fitted by the exponential function [42,43]

(8) $$D\left(t\right)=Ae^{-\frac{t}{3\lambda }}$$

where *A* is a function of elastic modulus and *λ* is the extensional relaxation time. By fitting data, the extensional relaxation time is calculated to be 80.91 ms for 10% PVDF/DMF solution, 88.18 ms for 12% PVDF/DMF solution, and 96.90 ms for 14% PVDF/DMF solution. Figure 6 demonstrates a linear relation between relaxation time and polymer concentration. It indicates that high concentration of PVDF/DMF solution has long extensional relaxation time. 

Furthermore, the Hookean dumbbell model predicts that there is a critical extension rate ${\dot{\varepsilon }}_{c}$ = 1/2*λ* that indicates the viscoelastic effect in an extensional flow [44]. For $\lambda \dot{\varepsilon }$ < 1/2, the contraction force dominates and polymer molecule remains in a modestly deformed coiled state. While $\lambda \dot{\varepsilon }$ > 1/2, the drag force dominates and polymer molecule undergoes a coil-stretch transition and becomes highly extended. Here, the critical extension rate is 6.18 s^−1^ for 10% PVDF/DMF solution, 5.67 s^−1^ for 12% PVDF/DMF solution, and 5.16 s^−1^ for 14% PVDF/DMF solution. 

### 4.2. Extensional Viscosity and Shear Viscosity of PVDF/DMF Solution

**Figure 7:**
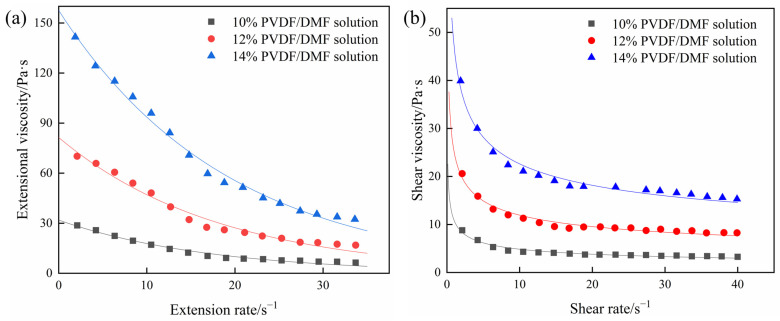
The viscosities of PVDF/DMF solutions: (**a**) extensional viscosity and (**b**) shear viscosity.

Once the pull force and the size of liquid column are measured, the first normal stress difference *N_1_* may be calculated by Equation (3). Consequently, the extension viscosity is available from the first normal stress difference divided by the extensional rate of liquid column by Equation (1). Figure 7a characterizes the extensional viscosities of 10%, 12%, and 14% PVDF/DMF solutions with extension rate in the range of 34 s^−1^. At low extension rate of $\dot{\varepsilon }$ = 2.0 s^−1^, measured extensional viscosities are 28.38 Pa·s, 72.93 Pa·s, and 147.90 Pa·s for 10%, 12%, and 14% PVDF/DMF solutions, respectively. The extensional viscosities then drop to 4.44 Pa·s, 12.68 Pa·s, and 26.83 Pa·s for 10%, 12%, and 14% PVDF/DMF solutions, respectively, at high extension rate of $\dot{\varepsilon }$ ≈ 34 s^−1^. 

The values of extensional viscosities may be fitted with an exponential model

(9) $$\mu_{E}=\mu_{0}e^{-\tau \dot{\varepsilon }},$$

where *μ*_0_ is the zero-extension viscosity and *τ* is the time constant. By fitting, the zero-extension viscosity *μ*_0_ is 31.88 Pa·s for 10% PVDF/DMF solution, 81.39 Pa·s for 12% PVDF/DMF solution, and 157.53 Pa·s for 14% PVDF/DMF solution at zero strain rate. The time constant *τ* is 0.058 s, 0.055 s, and 0.052 s for 10%, 12%, and 14% PVDF/DMF solutions, respectively. The results indicate that PVDF/DMF solutions are extension-thinning fluids whose extensional viscosity decays exponentially with increasing extension rate. 

Figure 7b demonstrates the shear viscosities of 10%, 12%, and 14% PVDF/DMF solutions with the shear strain rate in the range of 40 s^−1^. Measured shear viscosities are 8.48 Pa·s, 20.02 Pa·s, and 37.67 Pa·s for 10%, 12%, and 14% PVDF/DMF solutions, respectively, at low shear rate of $\dot{\gamma }$ = 2.0 s^−1^. The shear viscosities drop to 2.99 Pa·s, 7.65 Pa·s, and 14.58 Pa·s for 10%, 12%, and 14% PVDF/DMF solutions, respectively, at high shear rate of $\dot{\gamma }$ = 40.0 s^−1^. 

The values of shear viscosities conform to the power law fit by Equation (4). By fitting, the consistency coefficient *K* is 10.80 Pa·s for 10% PVDF/DMF solution, 25.01 Pa·s for 12% PVDF/DMF solution, and 46.94 Pa·s for 14% PVDF/DMF solution. The power-law index *n* is 0.65, 0.68, and 0.68 for 10%, 12%, and 14% PVDF/DMF solutions, respectively. It indicates that PVDF/DMF solutions are shear-thinning or pseudo-plastic fluids whose shear viscosity decreases with increasing shear rate. 

Figure 7 illustrates that PVDF/DMF solutions are not only extension shinning but also shear shinning. The rheological behavior of PVDF/DMF solutions may be accounted for by entangled molecular chains [45]. At low strain rate, entanglement nodes of molecular chains have enough time to be reconstructed after they are released. The molecular chains remain at curled state and viscous resistance to flow is very high. With increasing strain rates, reconstructed entanglement nodes decreases considerably and the viscosity drops rapidly. PVDF/DMF solution may behave as a typical pseudo-elastic fluid. On the contrary, there is little time to relax the stresses between entanglement nodes and polymer chains are highly extended at high strain rate. Extended molecular chains have low resistance to flow. Once strain rate is high enough for the stresses to destroy all the entanglement nodes, extensional or shear viscosity tends to constant value. PVDF/DMF solution then exhibits Newtonian-like fluid behavior. On the other hand, high polymer concentration may bring about much entanglement of polymer chains and consequently high viscosity. 

### 4.3. Trouton Ratio

The Trouton ratios of PVDF/DMF solutions may be evaluated by Equation (7). Substituting Equations (4) and (9) into Equation (7), we get

(10) $$T_{r}=\frac{\mu_{0}e^{-\tau \dot{\varepsilon }}}{K{\dot{\gamma }}^{n-1}}=\frac{\mu_{0}}{K}{\left(\sqrt{3}\dot{\varepsilon }\right)}^{1-n}e^{-\tau \dot{\varepsilon }}.$$

**Figure 8:**
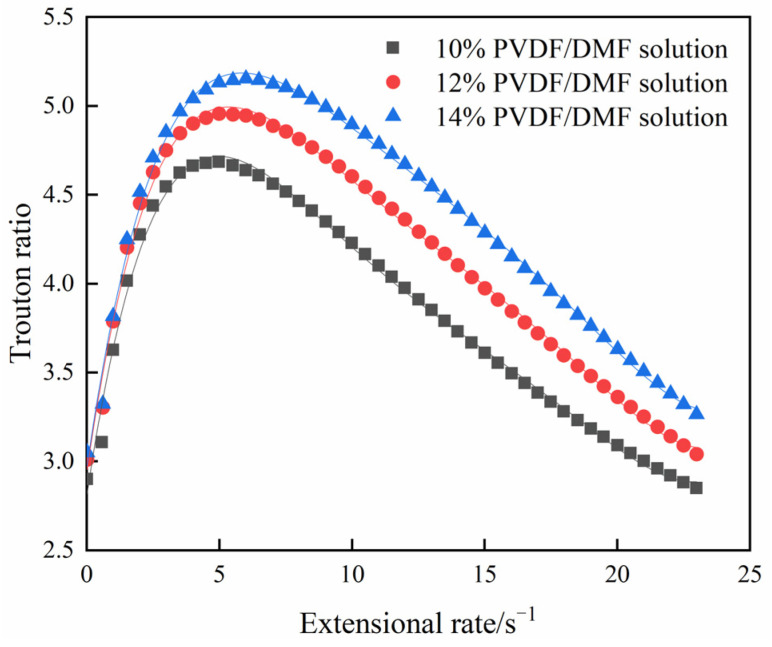
Trouton ratio versus extension rate.

Figure 8 characterizes the Trouton rations of PVDF/DMF solutions as the function of extension rate. At very low strain rate, the Trouton ratios are 2.86, 3.17, and 3.29 for 10%, 12%, and 14% PVDF/DMF solutions, respectively. All the values are close to three, which is consistent with basic property of polymer solutions. With increasing strain rate, the Trouton ratio rises steeply to a peak. The peak Trouton ratio is 4.70 at 6.0 s^−1^ for 10% PVDF/DMF solution, 4.96 at 6.0 s^−1^ for 12% PVDF/DMF solution, and 5.16 at 6.0 s^−1^ for 14% PVDF/DMF solution. Then, the Trouton ratio drops to 2.81 for 10% PVDF/DMF solution, 3.02 for 12% PVDF/DMF solution, and 3.26 for 14% PVDF/DMF solution at the extension rate of 23 s^−1^. 

The result shows that the Trouton ratio is not a monotonous function of strain rate although PVDF/DMF solutions are extension-thinning as well as shear-thinning. Thinning PVDF/DMF solutions exhibit that the decline rate of shear viscosity is higher than one of extensional viscosity below the strain rate corresponding to peak Trouton ratio and the other way around above the strain rate corresponding to peak Trouton ratio. The Trouton ratio somewhat shows the viscoelastic degree of polymer solutions. Elastic liquids are noted for high Trouton ratios. The maximum Trouton ratios of polymer melts may even reach several orders of magnitude. For dilute PVDF/DMF solutions in experiments, the peak Trouton ratio is less than 10, which represents low elastic effect in flows.

## 5. Conclusions

PVDF solutions are often applied to fabricate piezoelectric electronics recently by electrospinning. The electrospinning process stretches the liquid filament into nanofibers at very high stretch ratio and increases the polar *β* phase content in PVDF matter to improve the piezoelectricity. The solution forms a uniaxial extension flow during electrospinning under applied non-uniform electric forces. 

We investigate the extensional rheology of 10~14% PVDF/DMF solutions by using uniaxial extension flow as a viscometric flow. The 10~14% PVDF/DMF solutions exhibit an extension-thinning phenomenon in extension flows. The pull force as well as the size of liquid column decays exponentially with stretching time. Although PVDF/DMF solutions are also shear-thinning fluids, the Trouton ratio shows a characteristic peak of less than 10 in tests. Characteristic relaxation time is λ~100 ms and corresponding critical extension rate is ${\dot{\varepsilon }}_{c}$~5 s^−1^. Experimental results are very helpful to know the flow and deformation in electrospinning process of PVDF/DMF solutions. 

Presently, measured PVDF/DMF solution has a concentration of 10~14% while applied extension rate is less than 34 s^−1^. Further works are to test very dilute solutions with a high sensitive tension gauge at very low pull force and drive the extension flow with a high acceleration at very high strain rate. More efforts should be made to find a handy method for the measurement of transient extension viscosity. 

## Figures and Tables

**Table 1:** Material information of PVDF and DMF.

Chemical Name	Abbreviation	Chemical Structure	Purity	CAS Number	Molecular Weight (g/mol)	Supplier
Poly(vinylidene fluoride)	PVDF	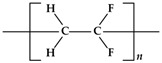	99%	24937-79-9	1,100,000	Arkema
N,N-dimethylformamide	DMF	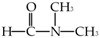	99.5%	68-12-2	73.9	Macklin

## Data Availability

The data that support the findings of this study are available from the corresponding author upon reasonable request.

## References

[1] Chola N.M., Sreenath S., Dave B. (2019). A non-gassing electroosmotic pump with sandwich of poly (2-ethyl aniline)-Prussian blue nanocomposite and PVDF membrane. Electrophoresis.

[2] Khalid H.U., Ismail M.C., Nosbi N. (2020). Permeation damage of polymer liner in oil and gas pipelines: A review. Polymers.

[3] Saxena P., Shukla P. (2021). A comprehensive review on fundamental properties and applications of poly(vinylidene fluoride) (PVDF). Adv. Compos. Hybrid Mater..

[4] Li B., Guo Z., Feng Y. (2021). A multi-functional photothermal-catalytic foam for cascade treatment of saline wastewater. J. Mater. Chem. A.

[5] Goldbach J.T., Amin-Sanayei R., He W., Ameduri B., Sawada H. (2016). Commercial synthesis and applications of poly(vinylidene fluoride). Fluorinated Polymers.

[6] Gardiner J. (2014). Fluoropolymers: Origin, production, and industrial and commercial applications. Aust. J. Chem..

[7] Wei Y., Zhou H., Deng H. (2022). “Toolbox” for the processing of functional polymer composites. Nanomicro. Lett..

[8] Dominick V., Rosato P.E. (2012). Plastics Processing Data Handbook.

[9] Liang Z.J. (2015). Elongation Rheology of Polymer Fluids.

[10] Sadeghi F., Tabatabaei S., Ajji A. (2009). Effect of PVDF characteristics on extruded film morphology and porous membranes feasibility by stretching. J. Polym. Sci. Part B Polym. Phys..

[11] Hedhli L., Mekhilef N., Moyses S. (2008). Characterization of randomly branched poly(vinylidene fluoride). Macromolecules.

[12] Guo Z., Nilsson E., Rigdahl M. (2013). Melt spinning of PVDF fibers with enhanced β phase structure. J. Appl. Polym. Sci..

[13] Bairagi S., Ali S.W. (2019). A unique piezoelectric nanogenerator composed of melt-spun PVDF/KNN nanorod-based nanocomposite fibre. Eur. Polym. J..

[14] He Z., Rault F., Lewandowski M. (2021). Electrospun PVDF nanofibers for piezoelectric applications: A review of the influence of electrospinning parameters on the β phase and crystallinity enhancement. Polymers.

[15] Cai X. (2017). A critical analysis of the α, β and γ phases in poly(vinylidene fluoride) using FTIR. RSC Adv..

[16] Ruan L., Yao X., Chang Y. (2018). Properties and applications of the β phase poly(vinylidene fluoride). Polymers.

[17] Kumarasinghe H., Bandara L., Bandara T. (2021). Fabrication of β-phase poly(vinylidene fluoride) piezoelectric film by electrospinning for nanogenerator preparations. Ceylon. J. Sci..

[18] Bera B., Sarkar M.D. (2017). PVDF based Piezoelectric Nanogenerator as a new kind of device for generating power from renewable resources. IOSR-JPTE.

[19] Gao K., Hu X., Dai C. (2006). Crystal structures of electrospun PVDF membranes and its separator application for rechargeable lithium metal cells. Mater. Sci. Eng. B-Adv..

[20] Yang C., Jia Z., Guan Z. (2009). Polyvinylidene fluoride membrane by novel electrospinning system for separator of Li-ion batteries. J. Power Sources.

[21] Liu K., Deng L., Zhang T. (2020). Facile fabrication of environmentally friendly, waterproof, and breathable nanofibrous membranes with high UV-resistant performance by one-step electrospinning. Ind. Eng. Chem. Res..

[22] Li Z., Zhu M., Shen J. (2020). All-fiber structured electronic skin with high elasticity and breathability. Adv. Funct. Mater..

[23] Xin Y., Zhu J., Sun H. (2018). A brief review on piezoelectric PVDF nanofibers prepared by electrospinning. Ferroelectrics.

[24] Wang X., Sun F., Yin G. (2018). Tactile-sensing based on flexible PVDF nanofibers via electrospinning: A review. Sensors.

[25] Du X., Zhou Z., Zhang Z. (2022). Porous, multi-layered piezoelectric composites based on highly oriented PZT/PVDF electrospinning fibers for high-performance piezoelectric nanogenerators. J. Adv. Ceram..

[26] Li J., Li Y., Lu Y. (2022). PDMS/PVDF Electrospinning Membranes for Water-in-Oil Emulsion Separation and UV Protection. Biomimetics.

[27] Lyu J.Y., Chen S., He W. (2019). Fabrication of high-performance graphene oxide doped PVDF/CuO/Al nanocomposites via electrospinning. Chem. Eng. J..

[28] Ng H.C.H., Corker A., García-Tuñón E. (2020). GO CaBER: Capillary breakup and steady-shear experiments on aqueous graphene oxide (GO) suspensions. J. Rheol..

[29] Yuan B., Ritzoulis C., Chen J. (2018). Extensional and shear rheology of a food hydrocolloid. Food. Hydrocoll..

[30] Ochowiak M., Broniarz-Press L., Rozanska S. (2012). The effect of extensional viscosity on the effervescent atomization of polyacrylamide solutions. J. Ind. Eng. Chem..

[31] Li B., Yu W., Cao X. (2020). Horizontal extensional rheometry (HER) for low viscosity polymer melts. J. Rheol..

[32] Hodder P., Franck A. (2005). A new tool for measuring extensional viscosity. Ann. Trans. Nord. Rheol. Soc..

[33] Sentmanat M.L. (2004). Miniature universal testing platform: From extensional melt rheology to solid-state deformation behavior. Rheol. Acta.

[34] Tirtaatmadja V., Sridhar T. (1993). A filament stretching device for measurement of extensional viscosity. J. Rheol..

[35] Luger H.J., Miethlinger J. (2019). Development of an online rheometer for simultaneous measurement of shear and extensional viscosity during the polymer extrusion process. Polym. Test..

[36] Jones D., Walters K., Williams P.R. (1987). On the extensional viscosity of mobile polymer solutions. Rheol. Acta.

[37] Różańska S., Różański J. (2017). Extensional flow of carboxymethylcellulose sodium salt measured on the opposed-nozzle device. Soft Mater..

[38] Coquand O., Sperl M. (2021). Rheology of granular liquids in extensional flows: Beyond the μ (I)-law. Phys. Rev. E.

[39] Haward S.J. (2014). Characterization of hyaluronic acid and synovial fluid in stagnation point elongational flow. Biopolymers.

[40] Kalimuldina G., Turdakyn N., Abay I. (2020). A review of piezoelectric PVDF film by electrospinning and its applications. Sensors.

[41] Abbas I., Hobiny A., Alshehri H. (2022). Analysis of Thermoelastic Interaction in a Polymeric Orthotropic Medium Using the Finite Element Method. Polymers.

[42] Dinic J., Zhang Y., Jimenez L.N. (2015). Extensional relaxation times of dilute, aqueous polymer solutions. ACS Macro Lett..

[43] Sousa P.C., Vega E.J., Sousa R.G. (2017). Measurement of relaxation times in extensional flow of weakly viscoelastic polymer solutions. Rheol. Acta.

[44] Larson R.G. (1999). The Structure and Rheology of Complex Fluids.

[45] Wang D.P., Zhao Z.H., Li C.H. (2021). Universal Self-Healing Poly (dimethylsiloxane) Polymer Crosslinked Predominantly by Physical Entanglements. ACS Appl. Mater. Inter..

